# Severe Acute Respiratory Distress in a Child with Hypereosinophilic Syndrome: A Case Report

**DOI:** 10.31729/jnma.8579

**Published:** 2024-05-31

**Authors:** Bipesh Kumar Shah, Shankar Prasad Yadav, Dheeraj Nagpal, Naveen Pokhrel, Samiksha Lamichhane

**Affiliations:** 1 Department of Pediatrics, B.P. Koirala Institute of Health Sciences, Dharan, Nepal; 2 Department of Radiodiagnosis, B.P. Koirala Institute of Health Sciences, Dharan, Nepal

**Keywords:** *case reports*, *children*, *hypereosinophilic syndrome*, *pulmonary infiltrates*, *steroids*

## Abstract

Hypereosinophilic syndrome with respiratory distress and multiorgan involvement is not so common in children. It is essential to identify this entity based on clinical, laboratory, and imaging features. Corticosteroids should be instituted at the earliest to stabilize the patient and prevent organ damage. Tropical infections are a common secondary cause in children warranting the administration of Diethylcarbamazine. We present a case of an adolescent male in respiratory distress with marked eosinophilia and organs involving the lungs (pulmonary infiltrates with effusion), heart (pericardial effusion), and abdomen (ascites with infiltrates in the liver) which was managed with steroids and anthelmintics. The case highlights the importance of identifying patients with Hypereosinophilic syndrome in pursuing thorough evaluation and commencing therapy.

## INTRODUCTION

Hypereosinophilic syndrome (HES) is characterized by an elevated absolute eosinophil count in peripheral blood >1500μL with organ or tissue involvement and severity ranging from benign to fatal.^1^ Primary HES includes eosinophilic myeloproliferative disorders involving mutations of tyrosine kinase receptor such as Platelet-derived growth factors A and B (PDGFR), Fibroblast growth factor receptor 1 (FGFR1) while secondary causes which are common in children include infectious (helminths, human immunodeficiency virus), atopic (asthma, allergic rhinitis), immunodeficiency disorders, rheumatological (Churg Strauss syndrome) and gastrointestinal diseases (Eosinophilic esophagitis).^2^ The drug history of antiepileptics (phenytoin, carbamazepine), and allopurinol should be considered in managing such cases as they present with marked eosinophilia.^3^

## CASE REPORT

An eleven years male, presented in a pediatric emergency with high-grade fever (104°F), cough, and fast breathing for 1 day. There was no history of loss of consciousness, abnormal body movement, abdominal distension, chest pain, or palpitation. One and half months back the child had cough, chest pain, and streaks of blood in the sputum for which he was admitted in nearby hospital; found to have pleural effusion which was treated with intravenous followed by oral antibiotics for couple of weeks; however no documents were available and the child remained asymptomatic until this presentation. The child belongs to a low middle-class family with a history of fishing in the local river, a history of roasted crab ingestion, and no history of atopy in the family. On examination the child was in severe distress (respiratory rate 35-40/min), maintaining oxygen saturation of 95 to 99% in a non-rebreathing bag at an oxygen flow of 6-8 liters per minute. There were extensive wheezes over the bilateral lung fields with crepitations over the left axillary area. The abdominal, cardiovascular and neurological examination were unremarkable.

In view of severe distress, the child was admitted to the pediatric intensive care unit with non-rebreather mask. The hemogram showed leukocytosis (19,410/cumm) with markedly elevated absolute eosinophil count (AEC) of 8734μL (reference:<650), hemoglobin of 11 gm/dl, platelet of 5.61 lacs/cu mm and elevated serum immunoglobulin E (6541 kUA/L, normal: <64) Renal and liver function tests were normal; blood & urine culture were sterile; HIV serology was nonreactive. Peripheral smear showed no cells with abnormal morphology, sputum for GeneXpert and mantoux was negative. Stool evaluation for worm infestation and blood evaluation for microfilaria were also negative.

**Figure 1:**
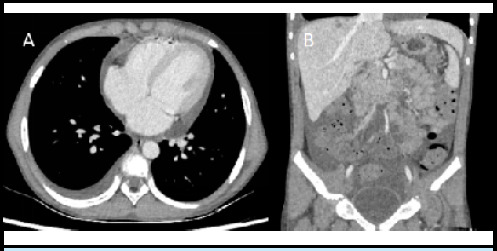
Contrast enhanced CT axial section (A) showing mild right pleural effusion and pericardial effusion. Coronal section (B) showing ascites in abdomen.

**Figure 2:**
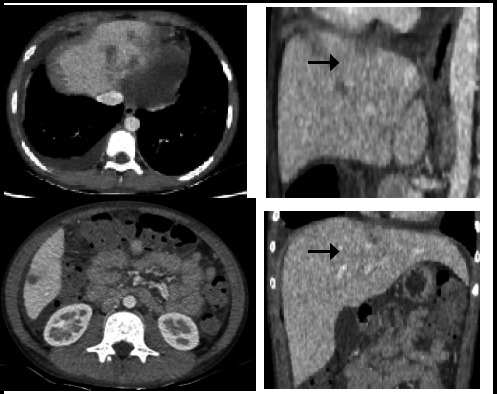
CT chest and abdomen showing right pleural effusion with pericardial effusion, ascites, and infiltrates in liver.

A high-resolution computed tomography (CT) of the chest and abdomen showed pulmonary infiltrates with mild right pleural effusion, mild pericardial effusion, infiltrates in the liver, and mild ascites (Figures 1 and 2).

Echocardiography was normal except for mild pericardial effusion. The other workups for dengue, rickettsial disease, and worm infestations (stool routine and microscopic for ova/cyst, serum filarial IgM and IgG) were normal. The child was managed initially with IV (intravenous) antibiotics piperacillin-tazobactam (300 mg/kg/day) and doxycycline (4.4 mg/kg/day) as child had history of previous admission and clinically suspected rickettsial infection. IV methylprednisolone 4 mg/kg/day in four divided doses along with Diethyl-Carbamazine (DEC) at 10 mg/kg/day in 3 divided dose was started on day 3 of admission. Albendazole (400 mg), a single dose was also given. The child started showing clinical improvement within a couple of days of starting methylprednisolone, which was switched to oral prednisolone at 2 mg/kg/day for 7 days. Antibiotics were given for a total of 10 days in view of suspected sepsis initially, DEC for 3 weeks, and steroids were tapered and stopped over one month. On follow-up after one month, the child was clinically stable with no signs of respiratory distress and AEC dropped to around 2000μL.

## DISCUSSION

There is a paucity of data in the pediatric cohort regarding hypereosinophilia syndrome.

Hypereosinophilic syndrome has varied manifestations, in a retrospective multi-center series, dermatologic manifestation (69%) was most common followed by pulmonary involvement in one-fourth of the patients, and cardiac & gastrointestinal involvement was present in 5 to 14%.^4^ Our case did not have dermal manifestation while pulmonary, cardiac, and liver involvement were present.

The larva gains access to pulmonary circulation from lymphatics inciting immunogenicity; antibody production and respiratory symptoms. The microfilariae could be isolated from the bronco-alveolar lavage brushings or lung biopsies.^5^ However, because of the limitation in performing bronchoscopy, the organisms couldn't be isolated. In tropical countries, the immune response to blood-borne microfilaria of *Wuchereia bancrofti* and less often *Brugia malayi,* can have similar symptoms. The testing for microfilaria was negative in our case.

The CT findings in acute eosinophilic pneumonia are ground-glass attenuation, inter-lobular septal thickening, broncho-vascular bundle thickening, and pleural effusion.^6^ These findings were reflected in our case. Liver involvement in HES is common and CT findings usually show lobar, segmental to subsegmental low-level attenuation due to eosinophilic infiltration.^7^

In a retrospective database, about 80% of the cases received steroids. The other forms of immunosuppressive used were hydroxyurea, interferon alfa, imatinib, and methotrexate.^8^ A total of 3 weeks of DEC (Diethylcarbamazine) is active against microfilaria causing tropical pulmonary eosinophilia.^5^

HES with life-threatening pulmonary manifestation is a very uncommon presentation, and at times poses diagnostic and management challenges. In developing countries, secondary causes like parasitic infections or drug exposure could be more common. The use of DEC, antihelminthics and steroids should not be delayed, while the workup for etiology should go parallelly.
